# The CCAAT‐binding complex mediates azole susceptibility of *Aspergillus fumigatus* by suppressing SrbA expression and cleavage

**DOI:** 10.1002/mbo3.1249

**Published:** 2021-11-22

**Authors:** Chi Zhang, Lu Gao, Yiran Ren, Huiyu Gu, Yuanwei Zhang, Ling Lu

**Affiliations:** ^1^ Jiangsu Key Laboratory for Microbes and Functional Genomics Jiangsu Engineering and Technology Research Center for Microbiology College of Life Sciences Nanjing Normal University Nanjing China

**Keywords:** *Aspergillus fumigatus*, azole resistance, CCAAT‐binding complex, protein cleavage, SrbA

## Abstract

In fungal pathogens, the transcription factor SrbA (a sterol regulatory element‐binding protein, SREBP) and CBC (CCAAT binding complex) have been reported to regulate azole resistance by competitively binding the TR34 region (34 *mer*) in the promoter of the drug target gene, *erg11A*. However, current knowledge about how the SrbA and CBC coordinately mediate *erg11A* expression remains limited. In this study, we uncovered a novel relationship between HapB (a subunit of CBC) and SrbA in which deletion of *hapB* significantly prolongs the nuclear retention of SrbA by increasing its expression and cleavage under azole treatment conditions, thereby enhancing Erg11A expression for drug resistance. Furthermore, we verified that loss of HapB significantly induces the expression of the rhomboid protease RbdB, Dsc ubiquitin E3 ligase complex, and signal peptide peptidase SppA, which are required for the cleavage of SrbA, suggesting that HapB acts as a repressor for these genes which contribute to the activation of SrbA by proteolytic cleavage. Together, our study reveals that CBC functions not only to compete with SrbA for binding to *erg11A* promoter region but also to affect SrbA expression, cleavage, and translocation to nuclei for the function, which ultimately regulate Erg11A expression and azole resistance.

## Graphical abstract

By directly interacting with the 34 *mer* promoter region of the azole target gene *erg11A*, the CCAAT binding complex (CBC) and SrbA co‐regulate the expression of Erg11A. In this study, we found that loss of HapB (a subunit of CBC) causes the upregulation of SrbA and SrbA cleavage‐related proteins: RbdB, SppA, and the Dsc complex. This facilitates SrbA cleavage and prolongs the nuclear retention of SrbA to further upregulate expression of Erg11A, suggesting a potential interplay exists between SrbA and CBC.

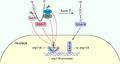

## 1 INTRODUCTION

To date, modern triazole antifungals, such as voriconazole (VRC), itraconazole (ITC) and posaconazole (POC), are still the most commonly used drugs for *A*. *fumigatus* infections (Fisher et al., 2018; Groll et al., 2017; Traunmuller et al., 2011). However, the emergence of azole‐resistant isolates resulting from continued azole stress or natural mutation leads to therapeutic failures and an increase in mortality (Chowdhary et al., 2017; Denning & Bromley, 2015; Snelders et al., 2008). Thus, understanding potential azole resistance mechanisms is key for developing new drugs and managing aspergillosis.

Previous studies have provided convincing evidence that azoles work as antifungals by targeting the key ergosterol biosynthesis enzymes lanosterol 14α‐demethylase Erg11 (also known as Cyp51) encoded by two homologous genes, *erg11A* (*cyp51A*) and *erg11B* (*cyp51B*), resulting in an accumulation of 14α‐methyl sterols (Becher & Wirsel, 2012; Georgopapadakou & Walsh, 1996; Joseph‐Horne et al., 1996). Excess 14α‐methyl sterols impair fungal growth by altering cell membrane permeability and stability (Cowen & Steinbach, 2008; Georgopapadakou & Walsh, 1996; Geria & Scheinfeld, 2008). Recent studies have proposed that azole antifungals also exert their fungicidal activity by triggering the synthesis of cell wall carbohydrate patches that penetrate the plasma membrane, thereby killing the fungus (Geissel et al., 2018). Investigation of azole‐resistant *A*. *fumigatus* suggests that the primary genetic alteration responsible for azole resistance is found within the *erg11A* locus (Burks et al., 2021). Among these azole‐resistant isolates, the substitution of leucine 98 for histidine (L98H) in the *erg11A* gene along with two copies of a specific 34‐bp tandem repeat (TR34) in the *erg11A* promoter (TR34/L98H) resulted in the overexpression of *erg11A*, which was found to be the predominant resistance mechanism (Chowdhary et al., 2017; Mellado et al., 2007; Snelders et al., 2008; Zhang et al., 2019). Understanding the regulatory mechanisms of *erg11A* expression can provide important insight into azole resistance mechanisms in fungal pathogens.

The expression of *erg11A* has been reported to be regulated by several transcription factors, including SrbA, AtrR, CBC (CCAAT‐binding complex), and NCT complex (negative cofactor two A and B), by directly binding to the promoter region of *erg11A* and consequently regulating azole susceptibility (Furukawa, Scheven, et al., 2020; Zhang et al., 2019). SrbA, a transcriptional regulator that belongs to the sterol regulatory element‐binding protein (SREBP) family, directly binds to the 34 *mer* of *erg11A* promoter and positively regulate *erg11A* expression to resist azoles (Bat‐Ochir et al., 2016; Blosser & Cramer, 2012; Willger et al., 2008, 2012). SrbA also binds to its own promoter to autoregulate its expression, as well as the promoters of sterol biosynthesis‐related genes in response to hypoxia (Blatzer et al., 2011; Chung et al., 2014). During hypoxia, endoplasmic reticulum (ER)‐associated full‐length SrbA undergoes protein cleavage involving the rhomboid protease RbdB, Dsc ubiquitin E3 ligase complex (DscA‐E), and signal peptide peptidase SppA, resulting in the release of the N‐terminal helix‐loop‐helix (HLH) transcription factor domain into the nucleus to function as a transcription factor (Bat‐Ochir et al., 2016; Dhingra et al., 2016; Vaknin et al., 2016; Willger et al., 2012). The nuclear translocation of the N‐terminal SrbA also occurs upon azole stress in *A*. *fumigatus* (Song et al., 2017). Consistent with the defective phenotypes observed in the *srbA* deletion mutant under hypoxia and azole conditions, *dscA*‐*E* null mutants show increased susceptibility to hypoxia and azole drugs (Dhingra et al., 2016; Willger et al., 2012). The Zn_2_‐Cys_6_ transcription factor AtrR has also been shown to be a positive regulator of *erg11A* in *A*. *fumigatus*. Similar to SrbA, the binding site of AtrR also falls in the 34 *mer* region of the *erg11A* promoter (Hagiwara et al., 2017; Paul et al., 2019). CBC, a heterotrimer composed of HapB, HapC, and HapE, is a negative regulatory complex of *erg11A* in *A*. *fumigatus*. CBC directly binds to the CGAAT motif within the 34 *mer* of the *erg11A* promoter, and CBC dysfunction increases *A*. *fumigatus* resistance to azoles (Gsaller et al., 2016). Notably, a clinically relevant HapE^P88L^ mutation in *A*. *fumigatus* is reported to significantly perturb the binding affinity of CBC to the *erg11A* promoter, resulting in an azole‐resistant phenotype (Camps et al., 2012; Hortschansky et al., 2017). Recently, the subunits of *A*. *fumigatus* NCT complex (negative cofactor two A and B), NctA and NctB, have been identified as a key regulator of azole resistance by directly binding to the TATA box‐like AT‐rich motifs within promoter regions of *erg11A*, *hapC*, *srbA* and *atrR* and regulating their expressions (Furukawa, van Rhijn, et al., 2020). Several studies have implicated that the molecular mechanism of azole resistance in *A*. *fumigatus* is highly complex and tightly regulated by a network of transcriptional activators and repressors. In this study, we analyzed an isolate 415‐2 selected from our previously reported azole‐resistant *A*. *fumigatus* library (Wei et al., 2017). Through next‐generation sequencing (NGS), we successfully identified a nonsense mutation in *hapB* conferring 415‐2 azole resistance. Based on a previous study showing that azole tolerance is governed by the opposing actions of SrbA and CBC on *erg11A* expression, we found that the increased expression of Erg11A and azole resistance induced by loss of HapB is dependent on SrbA. Additionally, we found that the lack of HapB not only increases SrbA expression but also promotes SrbA cleavage and nuclear translocation, which is probably due to the increased expression of RbdB, the Dsc complex, and SppA. These findings broaden our understanding of how SrbA and CBC coordinately regulate Erg11A expression and azole resistance and may provide a potential avenue for overcoming the resistance to azole drugs.

## 2 MATERIALS AND METHODS

### 2.1 Strains, media, and culture conditions

All *A*. *fumigatus* strains used in this study are listed in Table A1. In general, these strains were grown on solid minimal media (MM), which contained 0.02 g/ml agar, 0.01 g/ml glucose, 1 ml/L trace elements, and 50 ml/L 20× salt solution (Zhang & Lu, 2017). The liquid MM recipe does not contain agar. Uridine (5 mM) and uracil (10 mM) are required for uracil and uridine auxotrophic strains. To test the sensitivity of *A*. *fumigatus* to azole drugs, ITC and VRC were supplemented in MM or MM plus uridine and uracil (MMUU). For the plate assay, a 2 μl slurry containing 2 × 10^4^ spores was spotted onto solid MM at 37°C for 2 or 2.5 days. Longer culture time (4 days) was required for the observation of the growth phenotypes on MM with ITC or VRC.

### 2.2 Next‐generation sequencing analysis sequencing and single‐nucleotide polymorphism analysis

The fresh conidial spores of isolate 415‐2 were inoculated into liquid MM and shaken for 24 h at 37°C at 200 rpm, and the resulting mycelial pellets were dried and extracted to obtain genome DNA (gDNA). The next‐generation sequencing (NGS) experiment was performed at Shanghai OE Biotechnology Co., Ltd., as a commercial service. gDNA of 415‐2 was sequenced by using the Illumina HiSeq 2000 platform with 100‐bp paired‐end reads in a high‐output mode. An average depth of each nucleotide was gained. Sequence assembly and mapping were referred to the *A*. *fumigatus* A1163 genome (http://www.ncbi.nlm.nih.gov/assembly/GCA_000150145.1). Analysis of mapping quality and SNPs was performed by using a next‐generation sequencing data analysis suite, SHORE software.

### 2.3 Constructs for deletion, truncation, and GFP labeling strains

Strains (Δ*erg11A*, *hapB^165^*, Erg11A‐GFP, SppA‐GFP, RbdB‐GFP, and DscA/B/C/D/E‐GFP) were constructed at their native locus by our MMEJ‐CRISPR system as described previously (Zhang & Lu, 2017; Zhang et al., 2016). The sgRNA targeting the related gene was synthesized using the MEGAscript T7 Kit (Invitrogen). The corresponding repair templates with microhomology arms were obtained by PCR. Then, the repair template and sgRNA were transformed into a Cas9‐expressing *A*. *fumigatus* strain (ZC03/WT). WT^GFP−SrbA^, GFP‐SrbA^T^, SrbA^F^, and SrbA^T^ strains were constructed by transformation with plasmid prg3‐AMAI‐NotI (Aleksenko & Clutterbuck, 1997; Aleksenko et al., 1996). For the recycling usage of the selectable marker *pyr4*, 1 mg/ml 5‐FOA was used to screen recipient strains. For deleting *srbA*, the traditional homologous recombination strategy was employed. All primers are listed in Table A2. *A*. *fumigatus* transformation was carried out as previously described (Zhang & Lu, 2017; Zhang et al., 2016).

### 2.4 Molecular cloning

The plasmid p‐Ama1‐P_srbA_‐gfp‐srbA for labeling SrbA with GFP was constructed as follows: using primers Ama1‐srbA‐F and Ama1‐srbA‐R, the P_srbA_‐gfp‐srbA fragment containing the *srbA* promoter, GFP and *srbA* ORF was amplified from the gDNA of an N‐tagged GFP‐SrbA strain and then subcloned into the *BamH*I site of the plasmid prg3‐AMAI‐NotI, generating the plasmid p‐Ama1‐P_srbA_‐gfp‐srbA.

For colocalization analysis of GFP‐SrbA with the nucleus, the plasmid p‐Ama1‐P_srbA_‐gfp‐srbA‐P_gpdA_‐rfp‐H2A was generated as follows: the fragment P_srbA_‐gfp‐srbA was amplified from p‐Ama1‐P_srbA_‐gfp‐srbA with primers Ama1‐srbA‐F and gpd‐srbA‐R. The fragment P_gpdA_‐rfp‐H2A was amplified from pBARGPE‐P_gpdA_‐RFP‐H2A using primers gpd‐F and Ama1‐trpC‐R. Then, the two fragments were cloned into the *BamH*I site of the plasmid prg3‐AMAI‐NotI, yielding p‐Ama1‐P_srbA_‐gfp‐srbA‐P_gpdA_‐rfp‐H2A.

To analyze the localization of truncated SrbA (SrbA^T^, it contains residues 1 to 380 of the N‐terminus of SrbA), the plasmid p‐Ama1‐P_srbA_‐gfp‐srbA^T^‐P_gpdA_‐rfp‐H2A was generated as follows: The fragment P_srbA_‐gfp‐srbA^T^ was amplified from p‐Ama1‐P_srbA_‐gfp‐srbA with primers Ama1‐srbA‐F and Ama1‐srbA^T^‐R. The fragment P_gpdA_‐rfp‐H2A was amplified from pBARGPE‐P_gpdA_‐RFP‐H2A using primers gpd‐srbA^T^‐R and Ama1‐trpC‐R. Then, the two fragments were cloned into the *BamH*I site of the plasmid prg3‐AMAI‐NotI, yielding p‐Ama1‐P_srbA_‐gfp‐srbA^T^‐P_gpdA_‐rfp‐H2A.

The plasmid p‐Ama1‐P_srbA_‐srbA^F^/srbA^T^ was generated as follows: the fragment P_srbA_‐ srbA^F^/P_srbA_‐srbA^T^ was amplified from the gDNA of *A*. *fumigatus* with primers Ama1‐srbA‐F and Ama1‐srbA‐R/Ama1‐srbA^T^‐R. Then, the fragment was subcloned into the *BamH*I site of the plasmid prg3‐AMAI‐NotI, yielding p‐Ama1‐P_srbA_‐srbA^F^/srbA^T^.

The above plasmids were transformed into different background strains, which are listed in data Table A1.

### 2.5 Fluorescence microscopy

Fresh spores in 0.5 ml of liquid MM were grown under different treatment conditions (see legends) on sterile glass at 37°C after a set time. The coverslips with hyphae were gently washed with PBS buffer three times and then fixed with 4% paraformaldehyde. Then, hyphae were washed again with PBS. To detect nuclei, the hyphae were stained with Hoechst 33528 at a final concentration of 0.1 mg/ml for 30 min. The fluorescent images of the cells were directly captured with a Zeiss Axio Imager A1 microscope (Zeiss).

### 2.6 Quantitative real‐time PCR analysis

Fresh *A*. *fumigatus* conidia were grown in MM in a rotary shaker at 220 rpm at 37°C for 48 h. For measuring the relative mRNA expression levels of target genes, total RNA of related strains was extracted using the UNlQ‐10 Column TRIzol Total RNA Isolation Kit (Sangon Biotech, B511361‐0020), following the manufacturer's introduction. Then, cDNA synthesis was performed with the HiScript II Q RT SuperMix for qPCR Kit (Vazyme, R223‐01). For detecting the relative *srbA* gene copy number, the gDNA of *A*. *fumigatus* was extracted using Ezup Column Fungi Genomic DNA Purification Kit (Sangon Biotech, B518259‐0050). At least three biological replicates had been performed for each independent assay. The relative transcript levels of target genes and *srbA* gene copy number were calculated by the comparative threshold cycle (∆CT) and normalized against the expression of *tubA* mRNA level and *tubA* gene copy number, respectively. The difference of the relative mRNA expression and *srbA* gene copy number was determined as 2^−∆∆CT^. All the RT‐qPCR or qPCR primers and annotations are listed in data Table A2.

### 2.7 Western blotting

To extract GFP fusion proteins from *A*. *fumigatus* mycelia, 10^8^ conidia were inoculated into 100 ml of liquid MM under different treatment conditions (see legends) for a set time. Mycelia were collected, frozen in liquid nitrogen, and ground with a mortar and pestle. In general, protein extraction was performed using a previously described alkaline lysis strategy (Nandakumar et al., 2003). For extracting the nucleoprotein, the commercial nucleoprotein extraction kit (Beyotime, P0027) was used according to the manufacturer's instructions. The GFP fusion protein was detected by using an anti‐GFP mouse monoclonal antibody (Roche) at a 1:3000 dilution. Actin mouse monoclonal antibody (Proteintech, 66009‐1) at a 1:5000 dilution against actin was used as an internal loading control. Detailed procedures of protein extraction and western blotting were described previously (Nandakumar et al., 2003; Zhang et al., 2018; Zhang et al., 2016).

### 2.8 Recombinant CBC protein purification and electrophoretic mobility shift assay

To express His‐labeled CBC subunits *in vitro*, the exons of *hapB*, *hapC*, and *hapE* were amplified with three pairs of primers EmsA‐hapB‐F/EmsA‐hapB‐R, EmsA‐hapC‐F/EmsA‐hapC‐R, and EmsA‐hapE‐F/EmsA‐hapE‐R, respectively, and then ligated into the pET30a vector, subsequently transformed into BL21(DE3) Competent Cells were grown in LB medium at 37°C to an OD600 between 0.6 and 0.8, followed by addition of 0.1 mM isopropyl β‐D‐thiogalactoside. Protein purification was performed as previously described using a rapid Ni‐nitrilotriacetic acid (NTA) agarose minicolumn (Huang et al., 2015). EMSA was carried out according to previously described with minor modifications (Huang et al., 2015; Long et al., 2018). In briefly, each reaction contains consisted of 6 µl of 5× EMSA binding buffer, 1.5 µl of 1 mg/ml salmon sperm DNA (nonspecific competitor), 60 ng Cy5‐labeled probe (double‐stranded DNA), 0.5 µg HapB, 0.8 µg HapC, and 0.6 µg HapE. For competitive testing, a 30‐fold nonlabeled DNA probe (1.8 µg) as a competitive cold probe was added to the reaction. To confirm the specific binding of CBC to the binding motif CCAAT, the CCAAT motif within the promoter of the target gene was randomly mutated into a non‐CCAAT sequence. The reaction mixtures were incubated at 37°C for 0.5 h and then separated on a 5% polyacrylamide gel in 0.5× Tris‐borate EDTA buffer. Subsequently, the Cy5‐labeled probes were detected with an Odyssey machine (LI‐COR).

## 3 RESULTS

### 3.1 The azole resistance of isolate 415‐2 is due to the *hapB* mutation

**FIGURE 1:**
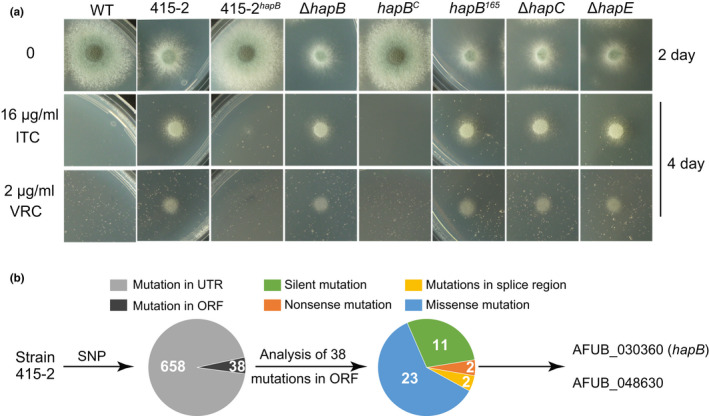
Dysfunction of the HapB/CBC complex leads to the resistance of 415‐2 isolate to azoles. (a) A series of 2 × 10^4^ conidia of wild‐type (A1160::*pyr4*/ZC03) 415‐2 isolate, 415‐2*^hapB^*, Δ*hapB*/*C*/*E*, *hapB^c^* ^,^ and *hapB^165^* strains were spotted onto MMUU (MM plus 5 mM uridine and 10 mM uracil) with different concentrations of ITC or VRC cultured at 37°C for 2 or 4 days. (b) The pie chart reflects the mutations in the 415‐2 genome by SNPs analysis

**FIGURE 2:**
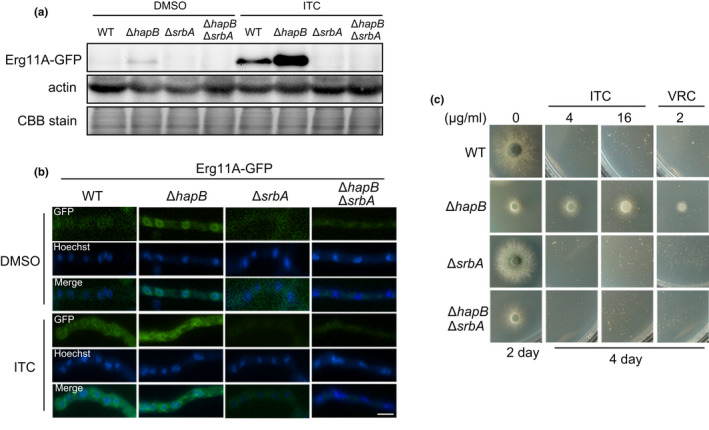
Increased expression of Erg11A and azole resistance induced by loss of HapB is dependent on SrbA. (a) Western blotting using a GFP antibody showed the protein expression levels of Erg11A‐GFP in the parental wild‐type, Δ*hapB*, Δ*srbA*, and Δ*hapB*Δ*srbA* strains. These strains were grown in 100 ml of MM at 37°C for 24 h, and then 0.2% DMSO or 16 μg/ml ITC was added to the media for 4 h. Immunoblot with an antibody against actin and Coomassie brilliant blue protein staining (CBB) of the total protein in SDS‐PAGE gels were used as two internal loading controls. (b) GFP signals of the fusion protein Erg11A‐GFP were observed in the indicated strains grown in MM at 37°C for 12 h and then shifted into MM with DMSO or ITC for 4 h. Hoechst was used to visualize the nuclei. The scale bar is 5 μm. (c) A series of 2 × 10^4^ conidia of the indicated strains were spotted onto solid MMUU with different concentrations of ITC and VRC cultured at 37°C for 2 or 4 days

Our previous study has obtained a library of azole‐resistant strains through a long‐term induction of azole treatment, however, the resistance mechanisms for a majority of these isolates have not been experimentally investigated (Wei et al., 2017). From this library, we found isolate 415‐2 without the mutations in *erg11A* showed high resistance to azoles. In comparison to the wild‐type strain, isolate 415‐2 displayed partial growth defects and high resistance to ITC and VRC (Figure1‐a). To identify the potential mutated genes leading to azole resistance, next‐generation sequencing (NGS) was implemented on isolate 415‐2. After BLAST analysis of the whole genomic sequence assembly based on the *A*. *fumigatus* A1163 genome, hundreds of single nucleotide polymorphisms (SNPs) were detected in the 415‐2 genome (Figure1‐b). Most of these SNPs were located in untranslated regions (UTRs), and 38 SNPs existed within the open reading frame (ORF), which contained 23 missense mutations, 2 nonsense mutations, 11 silent mutations, and 2 mutations in splice regions. Notably, one of the two nonsense mutations created a premature stop codon with the *hapB* gene at amino acid position 165 that led to the premature transcription termination of *hapB*. HapB is a subunit of CBC, which is a multimeric transcription factor complex comprising three subunits (HapB/HapC/HapE) and can bind to CC(G)AAT motif (Gsaller et al., 2016). Inactivation of the CBC by the absence of any of its subunits increases tolerance to different classes of drugs targeting ergosterol biosynthesis, including azoles, allylamines (terbinafine), and statins (simvastatin) (Gsaller et al., 2016). To test whether the azole resistance of isolate 415‐2 results from dysfunction of HapB/CBC, we transformed a wild‐type *hapB* gene into isolate 415‐2, generating 415‐2*^hapB^* strain. As shown in Figures 1‐a, 415‐2*^hapB^* exhibited similar colony growth and azole susceptibility to the wild‐type strain, demonstrating that the loss of HapB contributes to the azole resistance of 415‐2 isolate. Moreover, *hapB* truncation mutant that mimics the mutation in 415‐2 isolate (*hapB* ^165^) and the CBC deletion mutant (Δ*hapB*, Δ*hapC*, and Δ*hapE*) phenocopied the 415‐2 mutant with or without treatment of azole (Figure 1‐a), further demonstrating that the azole resistance of isolate 415‐2 is due to dysfunction of the HapB/CBC complex.

### 3.2 Increased expression of Erg11A and azole resistance induced by loss of HapB is dependent on SrbA

A previous study showed that CBC represses the mRNA expression of *erg11A* by binding the azole resistance‐associated 34 *mer* in the *erg11A* promoter (Gsaller et al., 2016). To further explore whether CBC and SrbA coordinately control Erg11A expression at the protein level, we labeled GFP at the C‐terminus of Erg11A in the wild‐type, Δ*hapB*, Δ*srbA*, and Δ*hapB*Δ*srbA* background strains. Immunoblotting analysis and fluorescence microscopic observation were performed to determine Erg11A‐GFP fusion protein expression in related strains in the presence or absence of ITC. Consistent with the increase in *erg11A* mRNA expression after treatment with azole drugs ITC and VRC (Blosser & Cramer, 2012; Du et al., 2021), the protein expression of Erg11A‐GFP also displayed a significant elevation after exposure to ITC for 2 h (Figure 2‐a). Especially, enhanced Erg11A‐GFP protein expression was much greater in Δ*hapB* than that in wild‐type under the ITC treatment condition. Interestingly, the protein expression of Erg11A‐GFP was almost completely suppressed in Δ*srbA* and Δ*hapB*Δ*srbA* compared to that of wild‐type strain irrespective of ITC treatment, confirming that SrbA is required for Erg11A protein expression. In line with immunoblotting results, fluorescence observation also showed that Erg11A‐GFP exhibited stronger GFP signals with the endoplasmic reticulum (ER)‐localized pattern in the Δ*hapB* strain than that in the wild‐type strain, and the GFP signals were barely observed in the Δ*srbA* and Δ*hapB*Δ*srbA* strains (Figure 2‐b). Moreover, the phenotypic analysis showed that Δ*hapB*Δ*srbA* presented a sensitive phenotype on ITC/VRC‐amended medium, which is similar to the Δ*srbA* strain (Figure 2‐c), suggesting that the increased expression of Erg11A and azole resistance induced by loss of HapB is dependent on SrbA.

### 3.3 The protein expression and localization of SrbA and HapB under azole treatment conditions

**FIGURE 3:**
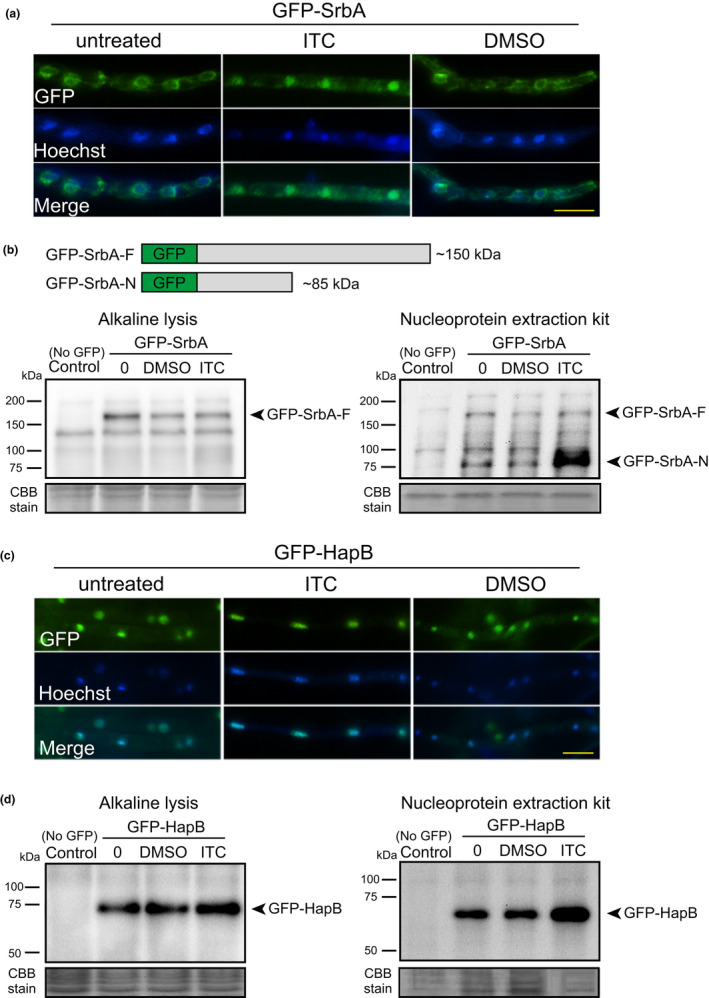
The protein expression and localization of SrbA and HapB. (a) and (c) The GFP‐SrbA and GFP‐HapB strains were grown in MM for 12 h and then shifted into MM with or without 0.2% DMSO or 16 μg/ml ITC for 2 h. The scale bar is 5 μm. (b) and (d) The indicated strains were grown in MM at 37°C for 24 h, and then 0.2% DMSO or 16 μg/ml ITC was added to the media for 2 h. The control “0” indicates the strains that were grown in MM for 26 h without DMSO or ITC treatment. The protein samples extracted using the alkaline lysis strategy or nucleoprotein extraction kit were examined by immunoblotting. The wild‐types strain without GFP tag was used as a control

**FIGURE A1:**
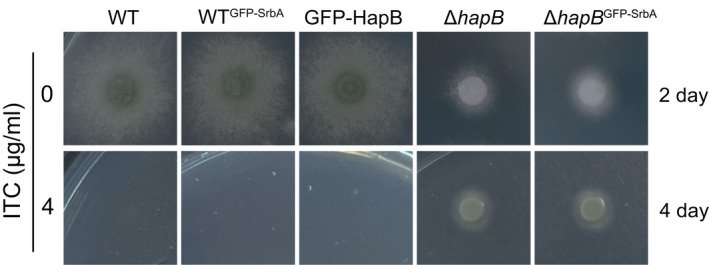
The growth phenotype of GFP‐tagged SrbA/HapB strains on MM with or without ITC stress. A series of 2 × 10^4^ conidia of wild‐type, WT^GFP−SrbA^, GFP‐HapB and Δ*hapB* and Δ*hapB* ^GFP−SrbA^ strains were spotted onto MM with or without ITC and cultured at 37°C for 2 or 4 days

Previous studies indicated that ER membrane‐bound SrbA can be cleaved at its C‐terminus and delivered into the nucleus to function as a transcription factor after hypoxia induction (Bat‐Ochir et al., 2016; Vaknin et al., 2016; Willger et al., 2012). The azole drug has a similar mechanism of action that triggers nuclear transport of SrbA to the nucleus (Song et al., 2017; Vaknin et al., 2016). To explore whether azole‐induced SrbA translocation was accompanied by the cleavage of SrbA, we generated the WT^GFP−SrbA^ strain by expressing the GFP‐SrbA fusion protein in the wild‐type background. The resulting strain exhibited similar growth phenotypes compared to the wild‐type strain under conditions with or without ITC treatment (Figure A 1), indicating that the N terminal GFP tag did not affect the function of SrbA. GFP signals were detected in the peripheral areas of the nucleus in WT^GFP−SrbA^ strain without ITC treatment, as indicated by the nuclear dye Hoechst (Figure 3‐a), indicating its localization in the ER. After ITC treatment for 2 h, GFP‐SrbA signals overlapped with the Hoechst signals, indicating the nuclear localization of SrbA. We confirmed that treatment with the DMSO control did not change the ER localization of GFP‐SrbA. To determine if SrbA cleavage was associated with its nuclear localization, total protein was extracted from the WT^GFP−SrbA^ strain using alkaline lysis followed by immunoblotting using a GFP antibody. Immunoblotting showed specific bands at approximately 150 kDa with or without ITC treatment, corresponding to the full‐length SrbA‐GFP fusion protein (hereafter named GFP‐SrbA‐F; GFP: ~27 kDa, SrbA: ~120 kDa), however, no cleaved SrbA bands were observed (Figure 3‐b). In contrast, the nuclear forms of SrbA (the cleaved N‐terminus, hereafter named GFP‐SrbA‐N) were clearly visible by using a specific nucleoprotein extraction kit (Figure 3‐b), suggesting that ITC induces SrbA protein cleavage and nuclear translocation. To explore the effects of ITC on HapB protein, the HapB was labeled with an N‐terminus GFP tag at its native locus. The GFP‐HapB fusion protein did not cause any morphological phenotypes compared to the parental strain (Figure A1), indicating that it is fully functional. As shown in Figure 3‐c, the GFP‐HapB fusion protein was constantly located in the nucleus, irrespective of ITC treatment, indicating that the localization of HapB was not affected by ITC. Notably, the immunoblotting analysis showed that ITC treatment could induce upregulation of GFP‐HapB expression (Figure 3‐d), suggesting that the nuclear‐localized HapB could respond to azole treatment.

### 3.4 The nuclear form of SrbA confers azole resistance and increases the expression of Erg11A in *A*.* fumigatus*

**FIGURE 4:**
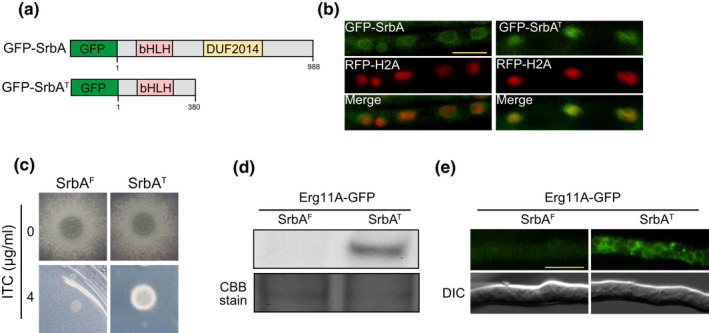
The nuclear form of SrbA renders *A*. *fumigatus* resistant to azole and increases the expression of Erg11A. (a) Prediction of a basic helix‐loop‐helix (bHLH) motif, transmembrane (TM), and DUF2014 domain in SrbA based on a SMART protein search (http://smart.embl‐heidelberg.de/) was performed, and a schematic is shown for the design of GFP‐SrbA and GFP‐SrbA^T^ constructs for detection of SrbA localization. (b) Colocalization analysis of GFP‐SrbA/GFP‐SrbA^T^ and RFP‐H2A was performed. The bar is 5 μm. (c) A series of 2 × 10^4^ conidia of SrbA^F^ and SrbA^T^ strains were spotted onto MM for 2.5 days and onto MM with ITC for 4 days. (d) Western blotting shows the protein expression of Erg11A in the SrbA^F^ and SrbA^T^ strains. (e) Fluorescence microscopy shows the GFP signals of Erg11A‐GFP in the SrbA^F^ and SrbA^T^ strains

Considering that ITC induces the SrbA cleavage and nuclear translocation, we wondered whether the nuclear form of SrbA was associated with azole resistance. To test this, we constructed a truncated SrbA strain (GFP‐SrbA^T^) that expresses a putative nuclear form of SrbA (SrbA^T^, which contains the first 380 aa of SrbA) fused with GFP at its N‐terminus under the control of the *srbA* native promoter in the wild‐type background (Figure 4‐a). To examine whether the GFP‐SrbA^T^ fusion protein localizes to the nucleus, we coexpressed RFP‐tagged histone H2A (RFP‐H2A) as a nuclear marker. As shown in Figure 4‐b, GFP signals of full‐length GFP‐SrbA were detected in the peripheral areas of RFP‐H2A, whereas GFP‐SrbA^T^ was colocalized with RFP‐H2A in minimal medium, indicating that the mutant with a nuclear form of SrbA was successfully constructed. To exclude the interference of the GFP tag with the function of SrbA^T^, we generated a SrbA^T^ strain that expresses SrbA^T^ without a GFP tag in the wild‐type background using the AMA1 vector. A SrbA^F^ strain expressing the full‐length of *srbA* was also constructed similarly as a control. To exclude the possibility that the AMA1 plasmids may introduce different *srbA* gene copies between SrbA^F^ and SrbA^T^ strains, we compared the relative gene copy number of *srbA* by quantitative real‐time PCR (qRT‐PCR) analysis. The result showed no significant difference in *srbA* copy number between SrbA^F^ and SrbA^T^ strains (Figure A 2‐a). The colony phenotype of SrbA^T^ strain was indistinguishable from SrbA^F^ strain on minimal medium but displayed increased resistance to ITC (Figure 4‐c), indicating the contribution of the nuclear form of SrbA to azole resistance. To verify whether expression of Erg11A could be changed in the SrbA^T^ strain, we expressed SrbA^T^ and SrbA^F^ in the Erg11A‐GFP labeled strain, respectively. As shown in Figure 4‐d, Erg11A‐GFP expression in the SrbA^T^ strain was significantly higher than that in the SrbA^F^ strain in minimal media. Fluorescence microscopy further confirmed that Erg11A‐GFP in the SrbA^T^ strain exhibited a strong ER‐localization signal, whereas in the SrbA^F^ strain, GFP fluorescence was barely observed (Figure 4‐e), suggesting that nuclear form of SrbA contributes to the increased expression of Erg11A, and the amount of nuclear form of SrbA in SrbA^T^ strain is greater than that in the SrbA^F^ strain. Taken together, these data suggest that the constitutive nucleus‐localized N‐terminus of SrbA renders *A*. *fumigatus* resistant to azole by upregulating Erg11A.

### 3.5 Lack of HapB increases SrbA expression and prolongs its nuclear retention

**FIGURE 5:**
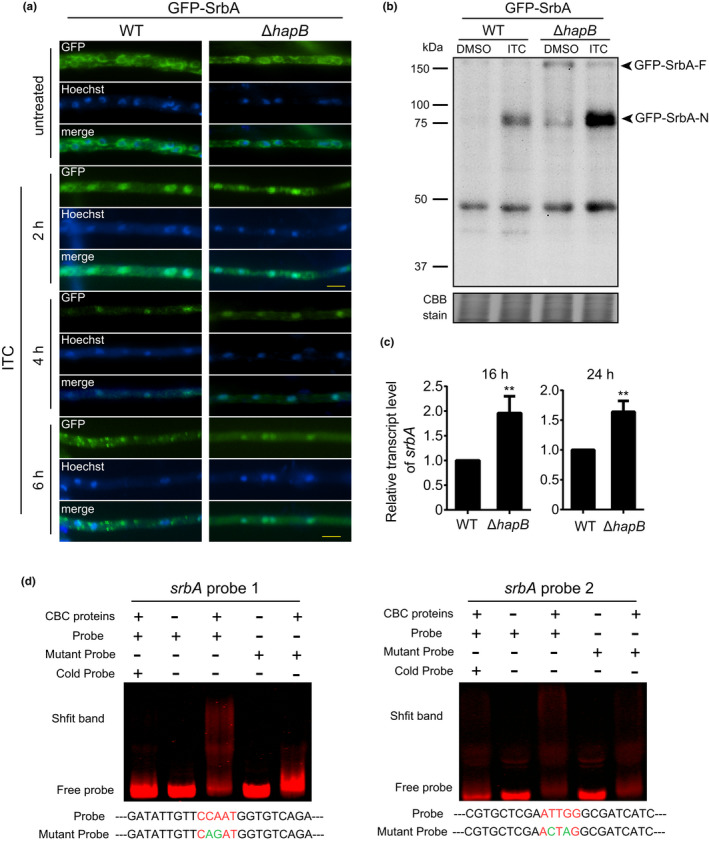
Lack of HapB prolongs nuclear retention of SrbA and increases the protein levels of the nuclear form of SrbA. (a) The WT^GFP−SrbA^ and Δ*hapB* ^GFP−SrbA^ strains were grown in MM for 12 h and then shifted into MM with 16 μg/ml ITC for 2, 4, and 6 h. The GFP signals were observed using a fluorescence microscope. The scale bar is 5 μm. (b) The indicated strains were grown in MM at 37°C for 24 h, and then 16 μg/ml ITC was added to the media for 4 h. The protein samples extracted by the nucleoprotein extraction kit were examined by immunoblotting. (c) The transcript levels of *srbA* in the wild‐type and Δ*hapB* strains grown in MM for 16 h and 24 h. Statistical significance was determined by Student's *t*‐test. **p < 0.01. Values are means ± SD from three independent replicates. (d) EMSA analysis of CBC binding to Cy5‐labeled promoter fragments of *srbA*. Two specific probes (1 and 2,) were designed for *srbA*. *srbA* probe 1/2 contains the CCAAT motif located at position 986/195 bp (−986/‐195) upstream of the *srbA* translational start site. The specificity of EMSA binding was validated by using a mutant probe or adding specific competitors/cold probe (unlabeled probe)

**FIGURE A2:**
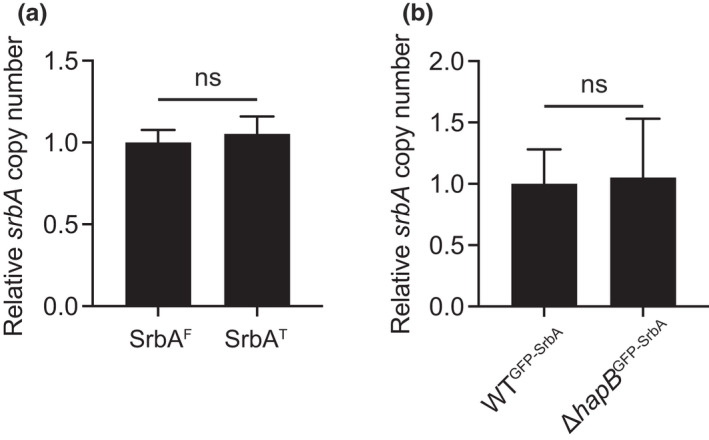
The relative *srbA* copy number in the related strains constructed by using the AMA1 vector. The related strains were grown in liquid MM with 220 rpm shaking at 37°C for 24 h. The respective genome was extracted from the resulting mycelium. Then qRT‐PCR was used to detect the relative *srbA* copy number, *tubA* gene was used as an endogenous control. Statistical significance was determined by Student's *t*‐test. ns, not significant. Values are means ± SD from three independent replicates

Based on the above findings, SrbA positively regulates Erg11A expression by its nuclear form, and overexpression of Erg11A is the main cause for azole resistance of the *hapB* deletion mutant. We next expressed the GFP‐SrbA by AMA1 vector in the Δ*hapB* and wild‐type background to examine whether a lack of HapB would affect the levels of the nuclear form of SrbA. The qRT‐PCR analysis showed no significant difference in *srbA* copy number between WT^GFP−SrbA^ and Δ*hapB* ^GFP−SrbA^ mutants (Figure A2‐b). Using fluorescence microscopy, we examined the localization of GFP‐SrbA in the Δ*hapB* ^GFP−SrbA^ and WT^GFP−SrbA^. As shown in Figure 5‐a, GFP‐SrbA showed ER‐localized patterns in the Δ*hapB* and the wild‐type strains when grown in minimal medium without ITC treatment and moved into the nucleus after ITC treatment for 2 and 4 h. Notably, with the prolongation of ITC treatment to 6 h, the nucleus‐localized fluorescence of GFP‐SrbA in the wild‐type strain showed the punctate GFP signals throughout the hyphae. In contrast, GFP‐SrbA in Δ*hapB* hyphae still exhibited a distinct nuclear localization pattern at the same time‐point, which suggests that the lack of HapB prolongs the retention time of GFP‐SrbA in the nucleus. Using immunoblotting with a GFP antibody, we examined the cleavage levels of SrbA in the Δ*hapB* and its parental wild‐type strains after ITC or DMSO treatment for 4 h. As shown in Figure 5‐b, the Δ*hapB* ^GFP−SrbA^ strain showed more accumulation of the nuclear forms of SrbA than the control strain WT^GFP−SrbA^, even under non‐induced conditions with DMSO treatment. In addition, we found that the Δ*hapB* mutant showed an increased protein level of SrbA compared to the wild‐type strain without ITC treatment, suggesting that HapB could be a negative regulator of SrbA. To confirm this, we conducted the qRT‐PCR analysis to compare the *srbA* transcription level in the wild‐type and Δ*hapB* mutant. As shown in Figure 5‐c, the mRNA level of *srbA* increased in the Δ*hapB* mutant. Previous studies have shown that CBC regulates the expression of downstream targets through binding to the 5′‐CCAAT‐3′ within their promoter regions (Gsaller et al., 2016; Hortschansky et al., 2017; Steidl et al., 2001). Sequence analysis revealed that two CCAAT motifs are located at position 849 and 195 base pairs (−849 and −195) upstream of the *srbA* translation start site. To further investigate whether CBC can directly bind to these two CCAAT motifs, we expressed and purified the HapB/C/E proteins in *Escherichia coli*, respectively, and then mixed them to form CBC complex for electrophoretic mobility shift assays (EMSA). As shown in Figure 5‐d, the *srbA* probe 1 (position: −849) mixed with CBC displayed a slow shift compared to the free probe without CBC treatment. Excess of unlabeled probe (cold probe) significantly reduced the binding activity of CBC with the Cy5‐labeled probe. Moreover, the mutated probe without the CCAAT motif showed a clear decrease in the binding activity of the upper complex. In comparison, *srbA* probe 2 (position: −195) also exhibited weak binding to CBC complex. These data suggested that the binding of CBC to the *srbA* promoter is dependent on the CCAAT motif.

### 3.6 Upregulation of SppA and the Dsc ubiquitin E3 ligase complex induced by the lack of HapB are responsible for proteolytic cleavage of SrbA

**FIGURE 6:**
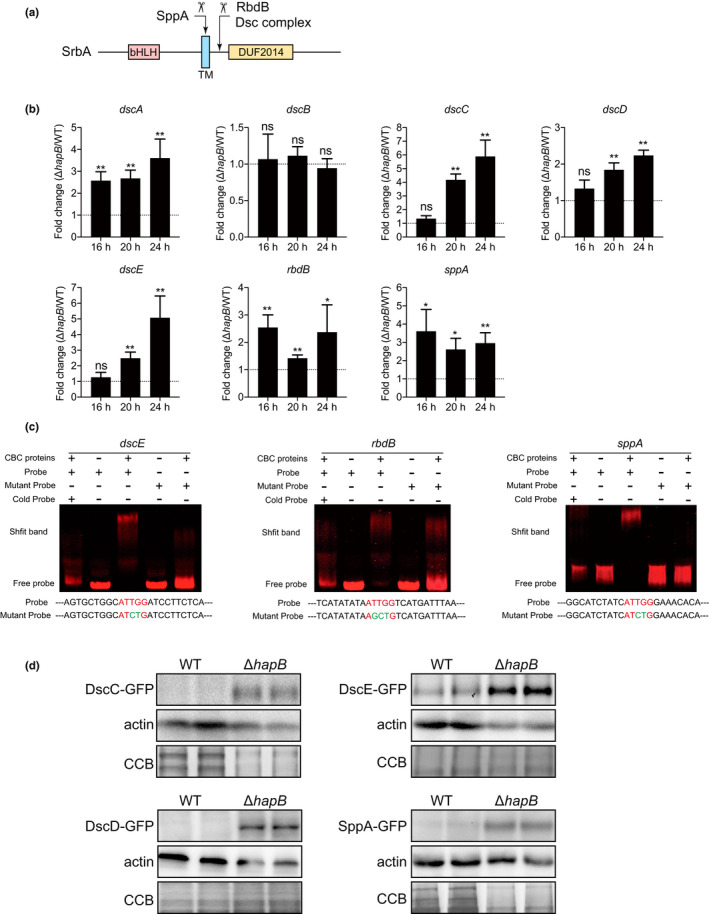
HapB regulates the expression of the proteins associated with the proteolytic cleavage of SrbA. (a) The putative schematic diagram of cleavage of *A*. *fumigatus* SrbA. (b) qRT‐PCR showed the mRNA expression of *dscA*‐*E*, *rbdB*, *sppA* in the wild‐type and Δ*hapB* backgrounds. The indicated strains were grown in MM at 37°C for 16, 20, and 24 h. Statistical significance was determined by Student's *t*‐test. *p < 0.05; **p < 0.01; ns, not significant. Values are means ± SD from three independent replicates. (c) EMSA analysis of CBC binding to Cy5‐labeled promoter fragments of *dscE*, *rbdB*, and *sppA*. Probes of *dscE*/*rbdB*/*sppA* contain the CCAAT motif located at −1467/‐600/‐170 position of their promoters, respectively. (d) Western blotting shows the protein expression of DscC‐E and SppA in the wild‐type and Δ*hapB* backgrounds. The indicated strains were grown in MM at 37°C for 24 h

**FIGURE A3:**
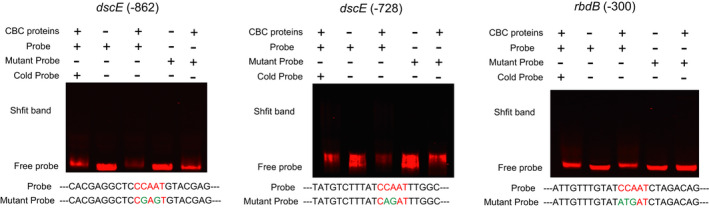
Electrophoretic mobility shift assays analysis of CBC binding to Cy5‐labeled promoter fragments of *dscE* and *rbdB*. The probe of *dscE* contains a CCAAT motif located at the −862/‐728 position of its promoter. The probe of *rbdB* contains a CCAAT motif located at the −300 position of its promoter

**FIGURE A4:**
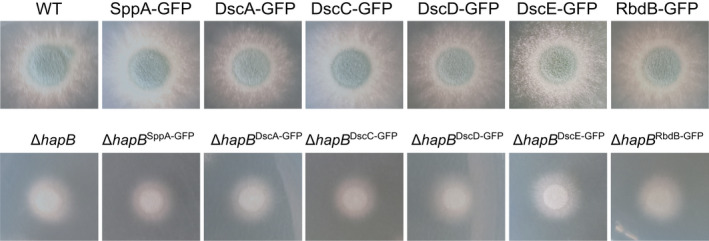
The growth phenotype of the GFP‐tagged SppA, DscA, DscC‐E, and RbdB strains on MM.

It has been reported that the cleavage of SrbA requires the rhomboid protease RbdB, the Dsc ubiquitin E3 ligase complex composed of five subunits (DscA‐E), and the signal peptide peptidase SppA, all of which are critical for translocation of SrbA from the ER into the nucleus and then SrbA could be transcriptionally activated (Figure 6‐a) (Bat‐Ochir et al., 2016; Dhingra et al., 2016; Vaknin et al., 2016; Willger et al., 2012). We wondered whether the increase in the nuclear forms of SrbA in Δ*hapB* might be related to the expression of RbdB, the Dsc complex, and SppA. We therefore performed the qRT‐PCR to detect the mRNA level of these genes. As shown in Figure 6‐b, except for *dscB*, the expression level of *rbdB*, *sppA*, and *dscA*/*C*/*D*/*E* was increased in the Δ*hapB* mutant compared to that of the wild‐type. As the sequence analysis showed that the promoter regions of all these upregulated genes contain at least one CBC binding motif CCAAT, we next selected *dscE*, *rbdB*, and *sppA* to perform EMSA analysis to test whether CBC binds to their promoters, the results showed that CBC can bind to the CCAAT motifs of *dscE* (position −1467), *rbdB* (position −600) and *sppA* (position −170) promoter regions with different degrees, but fails to bind other CCAAT regions of *dscE* (positions −728, −862) and *rbdB* (position −300) *in vitro* (Figure 6‐c and Figure A3). To further verify whether CBC could affect the protein expression of RbdB, SppA, and DscA/C/D/E, we tagged these proteins with GFP at the C‐terminus under the control of their respective promoters in the wild‐type and Δ*hapB* backgrounds, respectively. The phenotypic analysis confirmed that the tagged proteins are fully functional, as the resulting strains exhibited a similar growth phenotype to their respective parental strains (Figure A4). In line with the mRNA level, western blotting analysis showed that, except for the undetected DscA and RbdB expressions, DscC/D/E and SppA fusion proteins in the Δ*hapB* strain were increased compared to those in the parental wild‐type strain (Figure 6‐d), indicating that CBC represses the expression of SppA and the DscC/D/E at the protein level. Collectively, these results suggest that CBC represses the expression of SppA, RbdB, and the Dsc ubiquitin E3 ligase complex by binding the promoters of the corresponding genes.

## 4 DISCUSSION

In fungi, the transcriptional regulator SrbA and the CBC complex have been reported to transcriptionally regulate the expression of the azole target Erg11A and therefore play critical roles in azole resistance and sterol biosynthesis (Gsaller et al., 2016). It has been reported that both SrbA and CBC can bind to the TR34 region of the *erg11A* promoter, and they perform opposing actions to govern sterol biosynthesis and azole tolerance. The absence of any of the CBC subunits results in increased tolerance of *A*. *fumigatus* to azoles mainly due to the increased mRNA expression of *erg11A*. In contrast, the increased azole susceptibility in the *srbA* null mutant strain is the result of *erg11A* transcript insufficiency. In this study, we identified that the non‐*erg11A* azole‐resistant isolate 415‐2 harbors a mutation in the *hapB* gene that leads to the premature transcription termination of *hapB*. Importantly, we revealed a potential regulatory mechanism by which the CBC negatively regulates SrbA expression by directly binding to *srbA* promoter, and represses SrbA cleavage by down‐regulating the expression of the rhomboid protease RbdB, the Dsc ubiquitin E3 ligase complex, and the signal peptide peptidase SppA.

**FIGURE 7:**
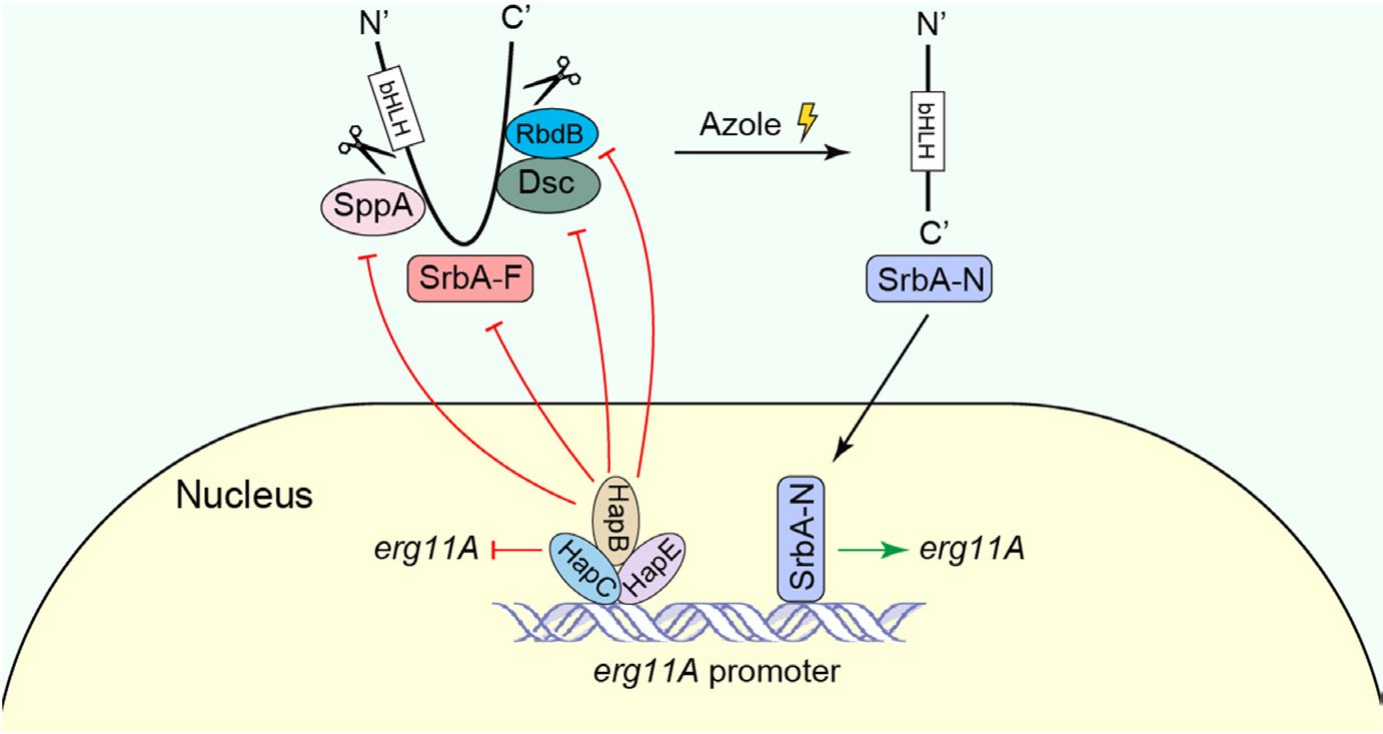
A proposed model is shown highlighting the mechanistic basis of *erg11A* expression regulated by CBC and SrbA. By directly interacting with the 34 *mer* region of the *erg11A* promoter, CBC and SrbA negatively and positively regulate the expression of *erg11A*, respectively. Upon azole stress, full‐length SrbA (SrbA‐F) is cleaved into the nuclear form (SrbA‐N) and subsequently transfers from the endoplasmic reticulum (ER) into the nucleus to exert its function. CBC not only represses the expression of *srbA* but also inhibits SrbA cleavage and nuclear translocation via repressing the expression of SrbA cleavage‐associated genes, including *rbdB*, Dsc complex genes, and *sppA*. These repressions are liberated in the mutants of CBC. Notably, CBC dysfunction also prolongs the retention time of SrbA‐N in the nucleus, which is not represented in the model

In mammals, low sterol activates ER‐localized SREBP by triggering its protein cleavage accompanied by its N‐terminal translocation to the nucleus (Bat‐Ochir et al., 2016; Brown & Goldstein, 1997, 1998, 1999). A similar mechanism exists in SREBP homolog, SrbA, in *Aspergillus* species under hypoxic conditions (Willger et al., 2012). We found that azole also induces the cleavage *of A*. *fumigatus* SrbA and its translocation into the nucleus (Figure 3‐a) (Song et al., 2017). In addition, expression of the putative nuclear forms of SrbA in the wild‐type strain increased Erg11A protein expression and tolerance to azole (Figure 4c‐e). These data suggest that azole exerts a similar influence on SrbA cleavage and nuclear translocation. The rhomboid protease RbdB, Dsc ubiquitin E3 ligase complex in *A*.* fumigatus*, and signal peptide peptidase SppA in *A*. *nidulans* are required for the cleavage of SrbA precursor protein (Bat‐Ochir et al., 2016; Dhingra et al., 2016; Willger et al., 2012). We found that the lack of HapB increased the nuclear forms of SrbA and prolonged the retention time of SrbA in the nucleus under azole treatment conditions (Figure 5a,b) may arise as a consequence of the upregulation of RbdB, SppA, and the Dsc complex (Figures 6‐b,d and 7). In addition, the HapB also negatively regulated the expression of SrbA, and loss of HapB increased the content of the total SrbA protein, which could also relatively increase nuclear forms of SrbA (Figures 5‐b and 7). According to previous reports, more than 2000 gene promoters are occupied by the CBC, and the high abundance of CBC binding motifs CC(G)AAT are identified in eukaryotic promoters (Furukawa, Scheven, et al., 2020), suggesting that CBC plays a role as a global regulator. Therefore, these data raise a possibility that HapB can negatively mediate the expression and cleavage of SrbA to regulate azole resistance besides repressing *erg11A* expression. In line with this hypothesis, our EMSA analysis validated that CBC can specifically bind to *srbA*, *dscE*, *rbdB*, and *sppA* *in vitro* via the conserved CCAAT motifs (*srbA*: position −986 and −195, *dscE*: position −1467, *rbdB*: position −600 and *sppA*: position −170). Previous ChIP‐seq analysis has also revealed that the CBC‐binding peaks were located at position 849 (−849) and 318 (−318) base pairs upstream of the *srbA* and *dscE* translational start sites, respectively (Furukawa, Scheven, et al., 2020), however, no typical CCAAT or CGAAT motif was observed in these regions. This may be because the ChIP peaks may result from the indirect binding events via intermediary partners, as the previous study showed that the transcription factor HapX forms a complex with CBC and CBC/HapX complex recognizes other DNA motifs than CC(G)AAT such as 5′‐RWT‐3′ and 5′‐TKAN‐3′ motifs (Furukawa, Scheven, et al., 2020). Nevertheless, our qRT‐PCR and western blotting experiments further showed that loss of CBC resulted in increased expression of SrbA and the majority of its cleavage‐related genes. In addition, since azole drugs and CBC deficiency have been reported to change the sterol profile (Gsaller et al., 2016; Shapiro et al., 2011), we therefore cannot rule out the potential role of altered sterol profile in the regulation of SrbA expression and nuclear retention, which needs further investigation and analysis.

## 5 CONCLUSION

In this study, our findings have revealed another plausible mechanism by which CBC dysfunction causes the upregulation of SrbA, and RbdB, SppA, and the Dsc complex facilitate SrbA activity, which ultimately elevates Erg11A expression and azole tolerance, and provides new insight into the molecular mechanism underlying the regulation of azole resistance. A working model summarizing the findings of this study is depicted in Figure 7.

## CONFLICT OF INTEREST

None declared.

## AUTHOR CONTRIBUTIONS

**Chi Zhang:** Conceptualization (lead); Data curation (lead); Formal analysis (lead); Investigation (equal); Methodology (equal); Resources (equal); Software (equal); Validation (equal); Writing‐original draft (equal). **Lu Gao:** Conceptualization (equal); Data curation (equal); Formal analysis (equal); Investigation (equal); Methodology (equal); Resources (equal). **Yiran Ren:** Conceptualization (equal); Methodology (equal). **Huiyu Gu:** Conceptualization (equal); Investigation (equal). **Yuanwei Zhang:** Formal analysis (equal); Funding acquisition (equal); Methodology (equal); Project administration (equal); Supervision (equal); Writing‐original draft (equal); Writing‐review & editing (equal). **Ling Lu:** Funding acquisition (equal); Investigation (equal); Methodology (equal); Project administration (equal); Resources (equal); Supervision (equal); Writing‐review & editing (lead).

## ETHICS STATEMENT

None required.

## Data Availability

The data that support the findings of this study are available in this published article and its appendices.

## 1

A series of 2 × 10^4^ conidia of related strains were spotted onto MM and cultured at 37°C for 2 days

**TABLE A1:** Strains used in this study

Strains	Genotype	Source
ZC03/WT	**Δ** *ku80; pyrG1; AMA1::P_gpdA_::Cas9::pyr4;*	Zhang et al., 2016
415‐2	Δ*ku80; pyrG1;* Δ*algA::pyr4; hapB^Q166^*	From Lu Lab
415‐2*^hapB^*	Δ*ku80; pyrG1;* Δ*algA::pyr4; hapB^Q165^; hapB::hph*	This study
Δ*hapB*	ZC03; Δ*hapB*::*hph*	Ren et al.,^2021^
*hapB^c^*	Δ*ku80; pyrG1;* Δ*hapB::hph; hapB::pyr4*	From Lu Lab
*hapB^165^*	ZC03; *hapB^Q165^*::*hph*	This study
Δ*hapC*	ZC03; Δ*hapC*::*hph*	Ren et al.,^2021^
Δ*hapE*	ZC03; Δ*hapE*::*hph*	Ren et al.,^2021^
Erg11A‐GFP	ZC03; *erg11A*‐*gfp*::*ptrA*	This study
Δ*hapB* ^Erg11A−GFP^	ZC03; Δ*hapB*::*hph*;*erg11A*‐*gfp*::*ptrA*	This study
Δ*srbA* ^Erg11A−GFP^	Δ*ku80; pyrG1; ΔsrbA::pyr4; erg11A‐gfp::ptrA*	This study
Δ*hapB*Δ*srbA* ^Erg11A−GFP^	Δ*ku80; pyrG1;* Δ*srbA::pyr4;* Δ*hapB::hph; erg11A‐gfp::ptrA*	This study
Δ*hapBΔerg11A*	ZC03; Δ*hapB*::*hph*;Δ*erg11A*::*ptrA*	This study
Δ*erg11A*	ZC03; Δ*erg11A*::*ptrA*	This study
Δ*srbA*	Δ*ku80; pyrG1;* Δ*srbA::pyr4*	This study
Δ*hapB*Δ*srbA*	Δ*ku80; pyrG1;* Δ*srbA::pyr4; hapB::hph*	This study
WT^GFP−SrbA^	Δ*ku80; pyrG1; AMA1::P_srbA_::gfp::srbA::pyr4*	This study
GFP‐HapB	ZC03; *gfp*::*hapB*; *hph*	Ren et al.,^2021^
GFP‐SrbA^RFP−H2A^	Δ*ku80; pyrG1; AMA1::P_srbA_::gfp::srbA::P_gpdA_::rfp::H2A::pyr4*	This study
GFP‐SrbA^T^ RFP‐H2A	Δ*ku80; pyrG1; AMA1::P_srbA_::gfp::srbA^1−380^::P_gpdA_::rfp::H2A::pyr4*	This study
SrbA^F^	Δ*ku80; pyrG1; AMA1::P_srbA_::srbA::pyr4*	This study
SrbA^T^	Δ*ku80; pyrG1; AMA1::P_srbA_::srbA^1−380^::pyr4*	This study
SrbA^T^ Erg11A‐GFP	Δ*ku80; pyrG1; erg11A‐gfp::ptrA; AMA1::P_srbA_::srbA^1−380^::pyr4*	This study
Δ*hapB* ^GFP−SrbA^	Δ*ku80; pyrG1;* Δ*hapB::hph; AMA1::P_srbA_::gfp::srbA::pyr4*	This study
SppA‐GFP	ZC03; *sppA*‐*gfp*::*ptrA*	This study
Δ*hapB* ^SppA−GFP^	ZC03; Δ*hapB*::*hph*; *sppA*‐*gfp*::*ptrA*	This study
DscA‐GFP	ZC03; *dscA*‐*gfp*::*ptrA*	This study
Δ*hapB* ^DscA−GFP^	ZC03; Δ*hapB*::*hph*; *dscA*‐*gfp*::*ptrA*	This study
DscB‐GFP	ZC03; *dscB*‐*gfp*::*ptrA*	This study
Δ*hapB* ^DscB−GFP^	ZC03; Δ*hapB*::*hph*; *dscB*‐*gfp*::*ptrA*	This study
DscC‐GFP	ZC03; *dscC*‐*gfp*::*ptrA*	This study
Δ*hapB^DscC^* ^−^ *^GFP^*	ZC03; Δ*hapB*::*hph*; *dscC*‐*gfp*::*ptrA*	This study
DscD‐GFP	ZC03; *dscD*‐*gfp*::*ptrA*	This study
Δ*hapB* ^DscD−GFP^	ZC03; Δ*hapB*::*hph*; *dscD*‐*gfp*::*ptrA*	This study
DscE‐GFP	ZC03; *dscE*‐*gfp*::*ptrA*	This study
Δ*hapB* ^DscE−GFP^	ZC03; Δ*hapB*::*hph*; *dscE*‐*gfp*::*ptrA*	This study
RbdB‐GFP	ZC03; *rbdB*‐*gfp*::*ptrA*	This study
Δ*hapB* ^RbdB−GFP^	ZC03; Δ*hapB*::*hph*; *rbdB*‐*gfp*::*ptrA*	This study

**TABLE A2:** Primers used in this study

Name	Sequence	Intention
sgRNA‐R	AAAAAAGCACCGACTCGGTGCC	for the DNA template of sgRNA
T7‐hapB‐sgRNA2‐F	TAATACGACTCACTATAGGGATAGTTGGGTGCCCATTGTTTTAGAGCTAGAAATAGC	for the DNA template of hapB‐sgRNA2 (RNA); for constructing *hapB^165^*
hapB^T^‐hph‐R	CTCATACGACGCTGTTGCATCTGGGCATTTTGGGTCGAGTGGAGATGTGGAGTGGG	for the repair template of *hapB* truncation
hapB^T^‐hph‐F	AGATTTCAGGACAGATG CCC ATG GGT ACA TAA	for the repair template of *hapB* truncation
hapB‐seq‐F	ACTCGAGCTGTTACTGGGTTAAG	diagnostic primer for *hapB^165^*
hapB‐seq‐R	ATGAGCCGATCGACAAGAGGGTA	diagnostic primer for *hapB^165^*
SrbAP1	AAATGGGTTCGTTGTATG	for deleting *srbA*
SrbAP2	TCTCCAGGTTGATATTGTTC	for deleting *srbA*
SrbAP3	CGATTAAGTTGGGTAACGCCACTTGACGGTGGAGTTAGA	for deleting *srbA*
SrbAP4	ATAAGTAGCCAGTTCCCGAAAGCACCAAGATGTGCCTCCAA	for deleting *srbA*
SrbAP5	GATGATGTCGGTAGGTGC	for deleting *srbA*
SrbAP6	GATCCCTCCATTCACCAG	for deleting *srbA*
Pyr4‐F	TGGCGTTACCCAACTTAATCG	for amplifying *pyr4*
Pyr‐R	GCTTTCGGGAACTGGCTACTTAT	for amplifying *pyr4*
SrbA‐F	AAACTCAACAAAGCGTCCAT	diagnostic primer for Δ*srbA*
SrbA‐R	CTGCGGGCAACATCATTC	diagnostic primer for Δ*srbA*
T7‐erg11A‐sgRNA‐F	TAATACGACTCACTATAGGGATTTGGTGTGATCGGAATGTTTTAGAGCTAGAAATAGC	for the DNA template of erg11A‐sgRNA (RNA); for deleting and tagging *erg11A*
erg11‐deletionptrA‐F	TCTAATCCTCGGGCTCACCCTCCCTGTGTCTCCTCGAAGCCTAGATGGCCTCTTGCATC	for the repair template of deleting and tagging *erg11A*
erg11‐deletionptrA‐R	CTCGAGGGGCTGAATTAAGTATAATACACCTATTCATGGCAGACACTGAAGCAAC	for the repair template of deleting *erg11A*
gfp‐erg11A‐F	CGGCTGGGAGAAGCGGTCGAAAAACACATCCAAGGGAGCTGGTGCAGGCGCTGG	for the repair template of tagging *erg11A*
erg11A‐seq‐F	CATTTCCCTCATCACTGCAACTC	diagnostic primer for Δ*erg11A* and *erg11A*‐*gfp*
nerg11A‐seq‐R	GTATAGGCAACAACACTTCAGGG	diagnostic primer for Δ*erg11A* and *erg11A*‐*gfp*
Ama1‐srbA‐F	CGGTTATGCCGTATGGATCCGATAGACTTCAGGTTAGTGATAGG	for amplifying P_srbA_‐gfp‐srbA fragment
Ama1‐srbA‐R	AATCAGCCTAGCTAGGATCCCTACGACTCATCGGAGAGC	for amplifying P_srbA_‐gfp‐srbA fragment
gpd‐srbA‐R	CGTCCGTCTCTCCGCATGCCTACGACTCATCGGAGAGC	for amplifying P_srbA_‐gfp‐srbA fragment (coupled with Ama1‐srbA‐F)
gpd‐F	GCATGCGGAGAGACGGACG	for amplifying P_gpdA_‐rfp‐H2A fragment
Ama1‐trpC‐R	AATCAGCCTAGCTAGGATCCCATGCATTGCAGATGAGCTG	for amplifying P_gpdA_‐rfp‐H2A fragment
Ama1‐srbA^T^‐R	AATCAGCCTAGCTAGGATCCCTATTCTGTGCCTAGGCCTTCAAG	for amplifying P_srbA_‐gfp‐srbA^T^/ P_srbA_‐srbA^T^fragment, coupled with Ama1‐srbA‐F
gpd‐srbA^T^‐R	CGTCCGTCTCTCCGCATGCCTATTCTGTGCCTAGGCCTTCAAG	for amplifying P_srbA_‐gfp‐srbA^T^ fragment (coupled with Ama1‐srbA‐F)
T7‐sppA‐sgRNA‐F	TAATACGACTCACTATAGGGAGTTCTTTCTTTCCTGTGTTTTAGAGCTAGAAATAGC	for the DNA template of sppA‐sgRNA (RNA); for tagging *sppA*
sppA‐gfp‐F	CAAGGCCAGCTTGTTTGGGTACATTGTCGGAAGGAGCTGGTGCAGGCGCTG	for the repair template of tagging *sppA*
sppA‐ptrA‐R	GTTCTCCTCTGAGGAGCGCAGTTCCCCACACTAGGAGATCGTCCGCCGATG	for the repair template of tagging *sppA*
sppA‐seq‐F	CCGTTAATGGTGACAGTCGCT	diagnostic primer for SppA‐GFP; qRT‐PCR primer
sppA‐seq‐R	GCACCTTGTGATTTGTCATCTG	diagnostic primer for SppA‐GFP
sppA‐RT‐R	GGAAAGGAAGATCAAATCGGAG	qRT‐PCR primer
T7‐dscA‐sgRNA‐F	TAATACGACTCACTATAGGGATGTTGTACGGACTGATGTTTTAGAGCTAGAAATAGC	for the DNA template of dscA‐sgRNA (RNA); for tagging *dscA*
dscA‐gfp‐F	CCCATTTGTCGGGAGTCAATCCCT CCTGTAGGAGCTGGTGCAGGCGCTG	for the repair template of tagging *dscA*
dscA‐ptrA‐R	TTTGTGTCCTGGGATGTTGTACGGACTGATCTAGGAGATCGTCCGCCGATG	for the repair template of tagging *dscA*
dscA‐seq‐F	GGTACGTGGCAACGTGCTG	diagnostic primer for DscA‐gfp; qRT‐PCR primer
dscA‐seq‐R	TCGCCAAGATCGAGGTCAGTG	diagnostic primer for DscA‐GFP
dscA‐RT‐R	CAGACTCCAGATCTGCCTCAGAC	qRT‐PCR primer
dscB‐RT‐F	CGGTATAGGATCAGCACCAGC	diagnostic primer for DscB‐gfp; qRT‐PCR primer
dscB‐RT‐R	AACTGCGACAGAGCCAGCTG	qRT‐PCR primer
T7‐dscC‐sgRNA‐F	TAATACGACTCACTATAGGGTGCCTTCCTTCAGCCATAGTTTTAGAGCTAGAAATAGC	for the DNA template of dscC‐sgRNA (RNA); for tagging *dscC*
dscC‐gfp‐F	GTTGCATTTGGCGCTATGCGCATTATGAATGGAGCTGGTGCAGGCGCTG	for the repair template of tagging *dscC*
dscC‐ptrA‐R	CTGGTCGCTTGGTGCCTTCCTTCAGCCATACTAGGAGATCGTCCGCCGATG	for the repair template of tagging *dscC*
dscC‐seq‐F	CGAAGACCGGTGGATGGATG	diagnostic primer for DscC‐GFP; qRT‐PCR primer
dscC‐seq‐R	CAGAGTGAAGGATATGTAGAC	diagnostic primer for DscC‐GFP
dscC‐RT‐R	CCTCAACCACATGGCACAGC	qRT‐PCR primer
T7‐dscD‐sgRNA‐F	TAATACGACTCACTATAGGGATCTCTTCATGCCAGTGTTTTAGAGCTAGAAATAGC	for the DNA template of dscD‐sgRNA (RNA); for tagging *dscD*
dscD‐gfp‐F	GTCGGGCCTGACGGTCGTGTTCAGCGGATACAATCCGGAGCTGGTGCAGGCGCTG	for the repair template of tagging *dscD*
dscD‐ptrA‐R	CCAACGGTGGATAGGATCTCTTCATGCCCTAGGAGATCGTCCGCCGATG	for the repair template of tagging *dscD*
dscD‐seq‐F	TGCCGGTGTAGGTGAACAGG	diagnostic primer for *dscD*‐*gfp*; qRT‐PCR primer
dscD‐seq‐R	GATGGTGCTGGTGAAGCTTC	diagnostic primer for DscD‐GFP
dscD‐RT‐R	CCGTCTGGTCCGAAGGTATG	qRT‐PCR primer
T7‐dscE‐sgRNA‐F	TAATACGACTCACTATAG GGTGTTGATAATGACATTAGTTTTAGAGCTAGAAATAGC	for the DNA template of dscE‐sgRNA (RNA); for tagging *dscE*
dscE‐gfp‐F	GACAGCGATGAGGAAGAGCAGGAAGTATCCGGAGCTGGTGCAGGCGCTG	for the repair template of tagging *dscE*
dscE‐ptrA‐R	TCGCCGGGGCAGTGCGCCTGTCTACCATAACTAGGAGATCGTCCGCCGATG	for the repair template of tagging *dscE*
dscE‐seq‐F	ACGACATTGCGCAATGGAAG	diagnostic primer for DscE‐GFP; qRT‐PCR primer
dscE‐yz‐R	CAGTCGGTACAGACGATCGAG	diagnostic primer for DscE‐GFP
dscE‐RT‐R	GCCCTGTGCAGCTTGCAGAAC	qRT‐PCR primer
srbA‐RT‐F	CCAGTCAGGACTGGACACAG	qRT‐PCR primer
srbA‐RT‐R	GGTGAGAAGTCTTTGTGCATCG	qRT‐PCR primer
tubA‐RT‐F	TGACTGCCTCCAGGGCTTCC	qRT‐PCR primer
tubA‐RT‐R	GCGTTGTAAGGCTCAACGAC	qRT‐PCR primer
rbdB‐RT‐F	CTACTGCTGGGCTCTGAAGC	qRT‐PCR primer, diagnostic primer for RbdB‐GFP
rbdB‐RT‐R	CGAGACCGAGAAGATAGCCT	qRT‐PCR primer
T7‐rbdB‐F	TAATACGACTCACTATAGGGAGGTAGGTTCAAGGACCGGTTTTAGAGCTAGAAATAGCA	for the DNA template of rbdB‐sgRNA (RNA); for RbdB‐GFP
rbdB‐gfp‐F	CTATCTGGGTACGAATCAGCGCCTCGGTCCTGGAGCTGGTGCAGGCGCTGG	for the repair template of tagging *rbdB* (RbdB‐GFP)
rbdB‐ptrA‐R	CATCGGCGGACGATCTCCTAGACCTACCTCGCTCAGCAGGGAGAGATG	for the repair template of tagging *rbdB* (RbdB‐GFP)
rbdB‐seq‐R	CCATGCCTAGTGTGGTAGTG	diagnostic primer for RbdB‐GFP
Pet30A‐F	GAATTCGAGCTCCGTCGACACCAGCTTGCGGCCGCACTCGAG	Linearization of Pet30A
Pet30A‐R	CATATGTATATCTCCTTCTTAAAGTTAAAC	Linearization of Pet30A
EmsA‐hapB‐F	GAAGGAGATATACATATGATGGAATACCCTCCACAATATCAAC	Amplification of *hapB* cDNA
EmsA‐hapB‐R	GTCGACGGAGCTCGAATTCACCATCTTCATCGGTTGGTTC	Amplification of *hapB* cDNA
EmsA‐hapC‐F	GAAGGAGATATACATATGATGTCGGCCTCTCCCTCGAAAG	Amplification of *hapC* cDNA
EmsA‐hapC‐R	GTCGACGGAGCTCGAATTCGTACGAGTCGCCTCCTGCTC	Amplification of *hapC* cDNA
EmsA‐hapE‐F	GAAGGAGATATACATATGATGGAACAGTCTTCGCAGAGCAC	Amplification of *hapE* cDNA
EmsA‐hapE‐R	GTCGACGGAGCTCGAATTCAGAAGAGTTTTGGCATACCTGC	Amplification of *hapE* cDNA
EmsA‐srbA2‐F	AGATGCAGCTAGCACGCCAGGTTGATATTGTTCCAATGGTGTCAGATACAGATACTTC	for *srbA* probe 2
EmsA‐srbA2‐MF	AGATGCAGCTAGCACGCCAGGTTGATATTGTTCAGATGGTGTCAGATACAGATACTTC	for *srbA* mutant probe 2
EmsA‐srbA2‐R	AGATGCAGCTAGCACGCTGTCGAAGCCGTTTCATCT	for *srbA* probe 2, for *srbA* mutant probe 2
EmsA‐srbA1‐F	AGATGCAGCTAGCACGCTGATTAGCGTGCTCGAATTGGGCGATCATCAATCATCGT	for srbA probe 1
EmsA‐srbA1‐MF	AGATGCAGCTAGCACGCTGATTAGCGTGCTCGAACTAGGCGATCATCAATCATCGT	for *srbA* mutant probe 1
EmsA‐srbA1‐R	AGATGCAGCTAGCACGACCTGTCAAGACCTGAGACG	for *srbA* probe 1, for *srbA* mutant probe 1
EmsA‐sppA‐F	AGATGCAGCTAGCACGCTTGTCTTGGCATCTATCATTGGGAAACACAGGGCTTTGAC	for *sppA* probe
EmsA‐sppA‐MF	AGATGCAGCTAGCACGCTTGTCTTGGCATCTATCATCTGGAAACACAGGGCTTTGAC	for *sppA* mutant probe
EmsA‐sppA‐R	AGATGCAGCTAGCACGGTTGCTCTGTGGTTGGAAATC	for *sppA* probe, for *sppA* mutant probe
EmsA−1467‐dscE‐F	AGATGCAGCTAGCACGGCCAGGAGTGCTGGCATTGGATCCTTCTCACTATTAGCAG	for *dscE* probe targeting −1467 region
EmsA−1467‐dscE‐MF	AGATGCAGCTAGCACGGCCAGGAGTGCTGGCATCTGATCCTTCTCACTATTAGCAG	for *dscE* mutant probe targeting −1467 region
EmsA−1467‐dscE‐R	AGATGCAGCTAGCACGCAAAAGCTGCAAATGCAGCTCC	for *dscE* probe and *dscE* mutant probe targeting −1467 region
EmsA−862‐dscE‐F	AGATGCAGCTAGCACGCATCCACGAGGCTCCCAATGTACGAGACCCTCTGTTCAG	for *dscE* probe targeting −862 region
EmsA−862‐dscE‐MF	AGATGCAGCTAGCACGCATCCACGAGGCTCCTAGTGTACGAGACCCTCTGTTCAG	for *dscE* mutant probe targeting −862 region
EmsA−862‐dscE‐R	AGATGCAGCTAGCACGAAGCCTGCTTTGGACGATTC	for *dscE* probe and *dscE* mutant probe targeting −862 region
EmsA−728‐dscE‐F	AGATGCAGCTAGCACGCTCAAGCCAAACTGACCTC	for *dscE* probe and *dscE* mutant probe targeting −728 region
EmsA−728‐dscE‐R	AGATGCAGCTAGCACGCGCTGATCCCCGCCAAATTGGATAAAGACATACCATGGCTTC	for *dscE* probe targeting −728 region
EmsA−728‐dscE‐MR	AGATGCAGCTAGCACGCGCTGATCCCCGCCAAATCTGATAAAGACATACCATGGCTTC	for *dscE* mutant probe targeting −728 region
EmsA−600‐RbdB‐F	AGATGCAGCTAGCACGATATATCTCATATATAATTGGTCATGATTTAATGAGCTAGAAG	for *rbdB* probe targeting −600 region
EmsA−600‐RbdB‐MF	AGATGCAGCTAGCACGATATATCTCATATATAAGCTGTCATGATTTAATGAGCTAGAAG	for *rbdB* mutant probe targeting −600 region
EmsA−600‐RbdB‐R	AGATGCAGCTAGCACGTGGGCTCTTCCAGTAACGCG	for *rbdB* probe and *rbdB* mutant probe targeting −600 region
EmsA−300‐RbdB‐F	AGATGCAGCTAGCACGCTTCTGCATTGTTTGTATCCAATCTAGACAGTGACACTGCAC	for *rbdB* probe targeting −300 region
EmsA−300‐RbdB‐MF	AGATGCAGCTAGCACGCTTCTGCATTGTTTGTATATGATCTAGACAGTGACACTGCAC	for *rbdB* mutant probe targeting −300 region
EmsA−300‐RbdB‐R	AGATGCAGCTAGCACGCGTGGTTTAATAATCAGTCAAG	for *rbdB* probe and *rbdB* mutant probe targeting −300 region
